# Endomyocardial fibrosis presented by ventricular tachycardia: case report

**DOI:** 10.1186/s43044-019-0027-x

**Published:** 2019-11-19

**Authors:** Mahmoud Abdelnabi, Abdallah Almaghraby, Yehia Saleh, Sherif Abd Elsamad, And Sara Elfawal

**Affiliations:** 1 Cardiology and Angiology Unit, Department of Clinical and Experimental Internal Medicine, Medical Research Institute, University of Alexandria, 165 El-Horeya Rd, Al Ibrahimeyah Qebli WA Al Hadrah Bahri, Qesm Bab Sharqi, Alexandria Governorate, Alexandria, 21561 Egypt; 2 Department of Cardiology, Faculty of Medicine, University of Alexandria, Alexandria, Egypt; 3 Michigan State University, East Lansing, MI USA; 4 Department of Radiology, Faculty of Medicine, University of Alexandria, Alexandria, Egypt

**Keywords:** Endomyocardial fibrosis, Ventricular tachycardia, Cardiac magnetic resonance, Echocardiography

## Abstract

**Background:**

Endomyocardial fibrosis (EMF) is a form of restrictive cardiomyopathy that is diagnosed mainly in children and young adults and is geographically found in Africa, Latin America, and Asia. It is a condition with high morbidity and mortality, unknown etiology, and no definitive treatment. Although its main clinical presentation is congestive heart failure with or without related supraventricular arrhythmia like atrial fibrillation, it very rarely presents with ventricular arrhythmias and tachycardias (VA, VT).

**Case presentation:**

We report a case of right ventricular (RV) EMF presented with recurrent attacks of hemodynamically unstable VT that required direct current (DC) cardioversion. The diagnosis was suspected by transthoracic echocardiography (TTE) and established by cardiac magnetic resonance (CMR). The patient underwent implantable cardioverter–defibrillator (ICD) implantation for secondary prevention of VT, and he was discharged safely on antiarrhythmic drugs with regular follow-up visits.

**Conclusion:**

EMF presenting with VT are quite rare and to the best of our knowledge, our case is the fourth case in the literature to report VT as a clinical presentation of EMF.

## Background

Endomyocardial fibrosis (EMF) is a progressive disease of unknown origin affecting children and young adults in African countries. Heart failure and supraventricular tachycardias are the main symptoms [1]. Ventricular arrhythmia (VT) is rarely encountered in EMF and only a few case reports exist [1, 2]. This report describes a very rare presentation of EMF with recurrent attacks of VT highlighting the previously published cases with such peculiar presentation.

## Case presentation

**Fig. 1:**
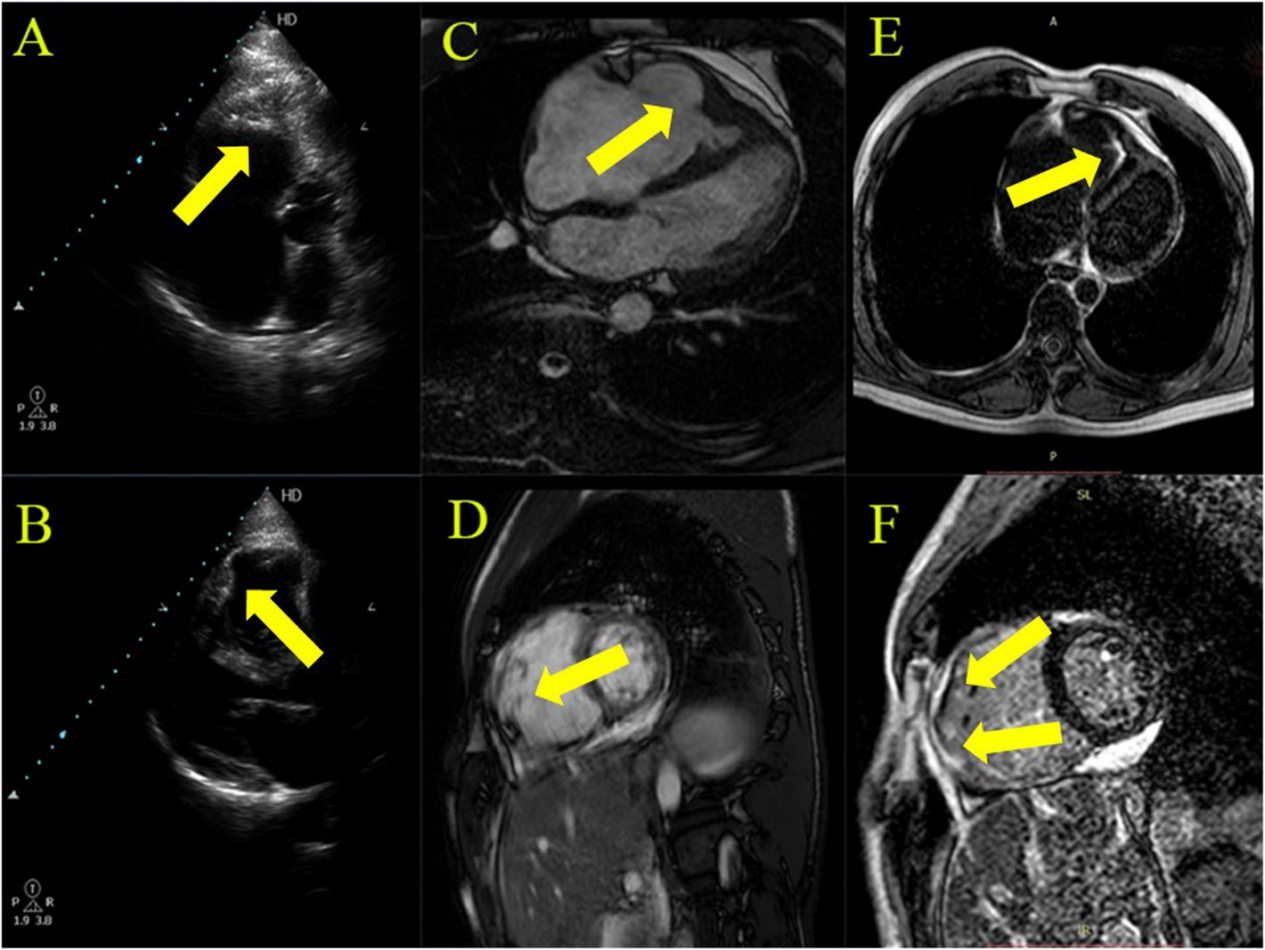
TTE and CMR of EMF. **a** 2D TTE, RV apical four-chamber modified view showed obliteration of the RV apex (marked with arrows). **b** 2D TTE, parasternal long axis view showing dilated RV dimensions (marked with arrows). **c**, **d** Cine CMR apical four-chamber and short axis views showing dilated RV dimensions (marked with arrows). **e**, **f** LGE CMR imaging showing subendocardial LGE enhancement at the RV apex and RV free wall (marked with arrows)


The case is a 45-year-old male patient with a past medical history of type 2 diabetes, no hypertension, and no history of cardiac illness. He started complaining of recurrent attacks of rapid regular palpitations 5 days prior to presentation. On the day of admission, the attack was persistent and associated with nausea and sweating. Upon examination, his blood pressure was unrecorded, his heart rate was 180 beats per minute, and his electrocardiogram (ECG) showed VT at 180 beats/minute with left bundle branch (LBBB) morphology and inferior axis. Urgent direct cardioversion (DC) was done, and he regained normal sinus rhythm 80 beats per minute with the right (RBBB), left axis deviation, and inverted T waves in right pericardial leads and became hemodynamically stable. Complete laboratory investigations including cardiac biomarkers and complete blood count (CBC) with no eosinophilia were unremarkable. Transthoracic echocardiography (TTE) showed a dilated right atrium and right ventricle with obliteration and retraction of the right ventricular (RV) apex and mild tricuspid valve regurgitation (Fig. 1a, b; Additional file 1: Video S1 and Additional file 2: Video S2) with preserved right and left ventricular systolic function and minimal pericardial effusion consistent with the diagnosis of RV EMF with a moderate severity (score of 10). Cardiac magnetic resonance (CMR) showed the right side with obliterated RV apex with subendocardial late gadolinium enhancement (LGE) consistent with the diagnosis of RV EMF (Fig. 1c–f; Additional file 3: Video S3 and Additional file 4: Video S4). An ICD was implanted for secondary prevention of VT, and he was discharged safely on amiodarone with regular follow-up visits.

**Additional file 1: Video S1.**




**Additional file 2: Video S2.**




**Additional file 3: Video S3.**




**Additional file 4: Video S4.**



## Discussion

EMF is a progressive disease that affects children and young adults in African countries with equal sex predilection [1]. Multiple factors have been implemented in the pathogenesis, such as eosinophilia, parasitic infections and environmental, genetic, and immunologic factors. Nonetheless, the exact etiology of EMF remains to be unknown. EMF is characterized by fibrous endocardial involvement of the inflow of the right or left ventricle or both and often involves in atrioventricular valves resulting in regurgitation [3]. Bi-ventricular disease occurs in about 50% cases with pure left ventricular affection in 40% and pure RV involvement in the remaining 10% of cases [1]. EMF is usually associated with heart failure symptoms and supraventricular tachycardias as the main symptoms.

As the ventricular endocardium develops fibrosis, significant diastolic dysfunction occurs. Subsequently, the atrium of the affected ventricle dilates causing atrial stretch and eventually patients develop supraventricular arrythmias. Atrial fibrillation is the most common arrythmia in EMF and it usually occurs in end-stage disease and predicts a poor prognosis. The exact mechanism of VT in EMF is still unknown. However, ventricular histopathology demonstrates increased type I collagen deposition, subendocardial infarction, and fibrosis which could explain why EMF can trigger ventricular arrythmias. Interestingly, in spite of the fibrous involvement of the myocardium in EMF, VT is extremely uncommon and only a few case reports exist [1, 2, 4]. The geographical distribution of the disease may be contributing as many of the cases are not diagnosed, and even if ventricular arrythmias occur, it is not reported. Prabhu et al. proposed that the involvement of a conducting system of the heart by the fibrotic process might result in bundle branch re-entry that can explain such a peculiar presentation [4]. Differential diagnosis of such condition includes idiopathic ventricular tachycardia, arrhythmogenic right ventricular dysplasia (ARVC), or right ventricular dilated cardiomyopathy. TTE has been and remains the main tool for diagnosis and follow up of all types of cardiomyopathy especially restrictive forms. EMF is diagnosed when there are two major or one major with two minor of the following criteria [5] (Table 1).

**Table 1:** Criteria for diagnosis and assessment of the severity of endomyocardial fibrosis [5]

Criterion	Score
Major criteria	
Endomyocardial plaques > 2 mm in thickness	2
Thin (≤ 1mm) endomyocardial patches affecting more than one ventricular wall	3
Obliteration of right ventricular or left ventricular apex	4
Thrombi or spontaneous echo contrast without severe ventricular dysfunction	4
Retraction of the right ventricular apex (right ventricular apical notch)	4
Atrioventricular-valve dysfunction due to adhesion of the valvular apparatus to the ventricular wall (the score is assigned according to the severity atrioventricular regurgitation)	1–4
Minor criteria	
Thin endomyocardial patches localized to one ventricular wall	1
Restrictive flow pattern across mitral or tricuspid valves	2
Pulmonary-valve diastolic opening	2
Diffuse thickening of the anterior mitral leaflet	1
Enlarged atrium with normal-size ventricle	2
M-movement of the interventricular septum and flat posterior wall	1
Enhanced density of the moderator or other intraventricular bands	1

A total score of < 8 = mild EMF; 8–15 = moderate EMF; > 15 = severe EMF

Nowadays, multi-modality cardiac imaging including CMR became a must to confirm the diagnosis of rare types of cardiomyopathies including EMF [6].

Given that the available literature is limited to case series that do not fully define treatment regimens, EMF is treated like other restrictive cardiomyopathies with diuretics and rate control for atrial fibrillation. In cases with advanced heart failure, endomyocardial resection with valve replacement or repair offered better long-term survival. However, high immediate postoperative mortality was reported. Moreover, given the lack of controlled studies, it is still not clear when is the appropriate timing of the surgery [1].

## Data Availability

The data is available for sharing.

## Abbreviations

ARVC: Arrhythmogenic right ventricular dysplasia
CMR: Cardiac magnetic resonance
DC: Direct current cardioversion
ECG: Electrocardiogram
EMF: Endomyocardial fibrosis
LBBB: Left bundle branch
LGE: Late gadolinium enhancement
RBBB: Right bundle branch
RV: Right ventricle
TTE: Transthoracic echocardiography
VA, VT: Ventricular arrhythmias and tachycardias
